# Fracture Resistance of Fiber-Reinforced Composite Restorations: A Systematic Review and Meta-Analysis

**DOI:** 10.3390/polym15183802

**Published:** 2023-09-18

**Authors:** Lorena Bogado Escobar, Lígia Pereira da Silva, Patrícia Manarte-Monteiro

**Affiliations:** FP-I3ID, Faculty of Health Sciences, University Fernando Pessoa, 4200-150 Porto, Portugal; 2022101334@ufp.edu.pt (L.B.E.); patmon@ufp.edu.pt (P.M.-M.)

**Keywords:** fiber-reinforced composites, glass fibers, polyethylene fibers, composite resin, fracture resistance, in vitro

## Abstract

Composite resin is universally used for posterior teeth restorations. Fibers have been suggested for the mechanical improvement of the restorations. This study assessed the fracture resistance of class II fiber-reinforced composite restorations and compared it with the fracture resistance of three control groups: (1) healthy teeth, (2) non-fiber-reinforced restorations and (3) unrestored cavities. A search was performed using PubMed, Web of Science and Google Scholar from 15 May to 12 June 2023. Only in vitro studies from the last 10 years were included for this systematic analysis. This study was registered in the PROSPERO database, it followed PRISMA guidelines and the risk of bias was assessed using the QUIN tool. Fracture resistance median values, in Newtons (N), were calculated for the experimental and control groups (95% confidence interval). For pairwise comparison, nonparametric tests (*p* < 0.05) were applied. Twenty-four in vitro studies met the inclusion criteria. The fracture resistance of the experimental group was 976.0 N and differed (*p* < 0.05) from all controls. The experimental group showed lower values of fracture resistance than healthy teeth (1459.9 N; *p* = 0.048) but higher values than non-fiber-reinforced restorations (771.0 N; *p* = 0.008) and unrestored cavities (386.6 N; *p* < 0.001). In vitro systematic outcomes evidenced that glass and/or polyethylene fibers improved the fracture resistance of composite restorations.

## 1. Introduction

Dental tissue loss affects the biomechanical behavior of the tooth and, inherently, the remaining teeth. Both extensive cavity preparations and restorations, and root canal treatments, can result in increased tooth structural fragility, which can lead to dental organ failure [1]. To minimize those effects, suitable restorative procedures are mandatory. Selection of the appropriate restorative material will depend on many conditions, such as the remaining tooth structure and the functional attributes to be returned to the organ, in addition to aesthetic considerations and the biological re-anatomization of structures [2].

Composed and complex cavity preparations of posterior teeth, due to the involvement of proximal walls, exhibit higher load concentration and greater cusp deflection and may have additional stress generated by the polymerization shrinkage of polymeric materials. According to their own cavity configuration, the number of walls and the marginal ridge loss, the extent of the occlusal isthmus and the depth of the preparation, the implicated tooth can become more susceptible to fracture [3].

Composite resin remains the material of choice for direct restorations of posterior teeth given their mechanical properties, aesthetics and clinical performance [4]. However, due to some limitations, over time adhesives and composites may compromise the biological, functional and clinical success of posterior teeth restorations, particularly regarding the detection of secondary caries lesions and the fracture of restorations and/or teeth [5]. In turn, clinical failure may occur due to inadequate fracture resistance of the polymeric material, or poor resistance to crack propagation, when the restorations are exposed to functional and para-functional mechanical loads [6,7].

Consequently, the tooth-strengthening effect of resin composites is still debated in the literature, especially when those medical devices are directly applied for the restoration of extensive tooth cavities in some intra-oral locations with high compressive and tensile loads [8]. In an attempt to improve the biomechanical behavior and durability of resin-composite restorations in posterior teeth, several mechanisms have been proposed, including the use of both fiber-reinforcement devices, applied directly and internally in cavity preparations, and polymeric materials [9].

Reinforcement by different types of fibers is not an innovative concept. It has been the basis of engineering and architecture in the construction of devices with high strength and fracture resistance. This resource being used for dental applications has been discussed in the literature since the early 1960s, when it was first proposed to reinforce acrylic denture bases [10].

Fiber-based devices can be used as a potential internal reinforcement for extensive direct resin-composite restorations of vital or endodontically treated teeth, of single crowns or fixed partial dentures and of direct root canal retentions. As external reinforcements, they are also a resource for tooth splinting in order to give some support needed by teeth due to periodontal or orthodontic conditions [11].

Structurally, fiber devices present three different components, the matrix or continuous phase, the fibers or dispersed phase, and the matrix/fiber interface. The reinforcement effect is based on a load–stress transfer from the polymer matrix to the fibers acting as a stress dissipator, internally strengthening the compromised tooth structure and serving as a fracture prevention layer when loads are applied [12].

The effectiveness of fiber reinforcement will depend on many variables such as the type of composite selected, the number of fibers in the resin matrix, the type, length, form, orientation (unidirectional, bidirectional, multidirectional) of the respective fibers, the adhesion to the polymer matrix and the resin impregnation of the fiber device [10].

Commonly, polyethylene and fiberglass are the most popular types of fibers used with direct composite restorations. Fiberglass is an inorganic material that varies according to its composition (A, C, D, E, R and S glass), presenting adequate aesthetics, high tensile strength, low thermal conductivity, high corrosion resistance and adequate surface chemistry, which allows its adhesion to the resin-based materials. However, it presents some limitations such as brittleness and low wear resistance [13]. Polyethylene fibers are constituted of aligned polymer chains with low density modulus, which enables higher impact strength. Ultra-high-modulus polyethylene fiber architecture allows uniform force distribution in more than one direction, with enhanced mechanical properties, high impact strength, excellent chemical resistance, low moisture absorption, vibration dampening ability and a low coefficient of friction [14].

Fibers associated with composites can potentially counteract the adverse effects of resins’ polymerization shrinkage and the consequent stress transferred to the composites and the remaining dental hard tissues. Simultaneously, they can promote improvement in the physical properties of composite restorations [15].

Considering clinical applications, this biomimetic approach may represent a less invasive and more conservative restorative option when compared to some polymers and restorative indirect techniques, as well as being more economical, more time efficient and a promising technique to prevent the fracture of extensive restorations in posterior teeth [16].

Several studies, mainly in vitro, have been conducted to evaluate the fracture resistance of fiber-reinforced composite resin restorations, but the results are still contradictory and controversial [17,18,19,20].

Several researchers have studied the failure mechanisms of synthetic fiber-reinforced composites by means of non-destructive evaluation techniques (NDE), since such techniques allow quality control and access to the whole structural integrity of materials. However, NDE techniques present some limitations such as the occurrence of several artifacts during data acquisition. Therefore, in order to obtain higher quality data, various filters and algorithms must be applied. Additionally, modelling and simulation of the failure mechanisms of fiber-reinforced composites reduce the number of experiments and, consequently, the costs [21]. Joffre et al. [22] and Strohrmann and Hajek [23] have validated the analysis of finite element models (FEMs) in order to study the failure mechanisms of different materials, finding a high level of agreement between their findings and experimental results.

Thus, it becomes critical to analyze the mechanical behavior of glass and/or polyethylene fibers regarding their reinforcement, or not, of composite resin restorations. Therefore, this systematic study aimed to evaluate the fracture resistance values of human posterior teeth extensively restored with fiber-reinforced composites, compared with those of healthy teeth, non-fiber-reinforced composite restorations and unrestored cavity preparations, by means of a quantitative analysis of in vitro outcomes.

For those purposes, the following null hypothesis was tested: the fracture resistance values of posterior teeth with fiber-reinforced composite restorations do not differ from those of healthy teeth or those of non-fiber-reinforced composite restorations or those of unrestored cavity preparations. 

## 2. Materials and Methods

This systematic study was registered (CRD42023425509) in the International Prospective Register of Systematic Reviews (PROSPERO) and followed the Preferred Reporting Items for Systematic Review and Meta-Analysis (PRISMA) guidelines [24,25]. The research question, based on the PICO model, was as follows: does the restoration of posterior teeth with fiber-reinforced composites show fracture resistance values similar to those of healthy posterior teeth, or non-fiber-reinforced composite restorations or unrestored extensive cavity preparations?

### 2.1. Inclusion Criteria, Exclusion Criteria and Eligibility

Only in vitro studies involving human permanent posterior teeth (molar, premolar or bicuspid) that tested the fracture resistance values of extensive class II, composed/complex, fiber-reinforced composite restorations, and compared those findings with the controls, healthy teeth, or with non-fiber-reinforced composites restorations, or with unrestored cavity preparations, were included. Also, only studies written in the English language and published in the last 10 years (from the year 2013 up to 12 June 2023) were scrutinized for this review.

To assess the fracture resistance values, in Newtons (N), of the vitro outcomes, studies were collected and analyzed according to the PICO strategy: *Problem*: human posterior teeth, after surgical exodontia procedure, with composed (2 surfaces) or complex (3 surfaces) cavity preparation, with or without root canal treatment; *Intervention*: direct resin-composite restorations reinforced with fiberglass and/or polyethylene fibers; *Comparison*: healthy teeth, non-fiber-reinforced composite restorations and unrestored cavity preparations in extracted posterior teeth; *Outcomes*: fracture resistance values (N).

Types of published research other than those considered in the inclusion criteria, such as those with a different methodology for the in vitro trials, those that assessed other mechanical properties of fiber-reinforced restorations, those that had incomplete abstracts or no full text, those that involved non-human teeth or other cavity preparation models, those with absence of a control group in the study protocol or with experimental groups that assessed root canal retentions, and even in vitro studies with other statistical models for composite analysis, were excluded. 

### 2.2. Search Strategy

#### 2.2.1. Sources of Information and Search Terms 

A methodical search was performed by two team members (L.B.E. and L.P.S.) using PubMed, Web of Science and Google Scholar from 15 May up to 12 June 2023. The search strategy included the terms “molar” OR “Premolar” OR “Bicuspid” OR “Posterior teeth” AND one of each of the 4 main Medical Subject Headings (MeSH), “fiberglass”, “polyethylene”, “composite resins”. The following terms, from tree structures, “fiber-reinforced”, “fiber-reinforced composite dentistry”, “fiber-reinforced restoration”, “fiber-reinforced composite resin”, “fiber cavity reinforcement”, “glass fibers”, ”polyethylene fibers”, “fracture resistance”, “fracture strength”, “fracture resistance fiber-reinforced composite” and “in vitro”, were joined by Boolean operators (“OR” and “AND”) according to the search question relevance. Search terms were included in the title and/or in the abstract and were appropriately modified for each database. The search strategy is presented in Table 1.

#### 2.2.2. Study Screening and Selection

Articles identified using the search terms were exported to Mendeley Desktop Reference Manager v2.94.0 software to check for duplicates. A first screening of record titles and abstracts was carried out by two independent examiners (L.B.E. and P.M.M.), considering the inclusion and exclusion criteria, the purposes of this research and the PICO approach. The remaining studies were assessed for eligibility, inclusion and exclusion criteria and qualitative synthesis by means of full-text screening. An identification number was assigned to each eligible study.

#### 2.2.3. Study Data

Bibliometric analysis was performed, recording the authors and year of publication. The methodology of examination included the in vitro studies’ aims, materials and methods, and their main findings in terms of the independent variables, the fracture resistance mean values and the respective standard deviations (expressed in Newtons) of all experimental and control groups.

### 2.3. Risk of Bias of Each Individual In Vitro Trial

The risk of bias was tested for each in vitro study using the Quality Assessment Tool for In Vitro Studies, QUIN [26], which consists of 12 criteria with scores for each domain (adequately specified = 2 points; inadequately specified = 1 point; not specified = 0 points; not applicable = excluded criteria). The final score for each study was obtained using the formula: total score × 100/2 × number of applicable criteria; a value that allows the classification of studies >70% = low risk of bias, 50% to 70% = medium risk of bias, and <50% = high risk of bias.

### 2.4. Data Synthesis and Statistical Analysis

Data were analyzed using the statistical software program IBM SPSS Statistics version 26. The degree of confidence was 95% in all tests (alpha equal to 0.05). For inferential analysis, the samples’ amplitude, dispersion and normality were explored using histograms complemented by the Kolmogorov–Smirnov test. The central point with the highest number of occurrences, the median, maximum and minimum values, and the respective quartiles (Q_1_–Q_3_) were determined. Fracture resistance median values were assessed for the experimental group (fiber-reinforced composite restorations) and for the three control groups (healthy teeth, non-fiber-reinforced composite restorations and unrestored cavity preparations) with a 95% confidence interval. For pairwise comparison of discrete numerical independent variables, within each group (experimental and control groups) and also between the experimental and the control groups, nonparametric tests, with a threshold of statistical significance for *p* < 0.05, were applied.

Several statistical models can be used for the statistical analysis of composites. According to Slah et al. [27], experimental approaches are insufficient when testing and analyzing the wear behavior of composite materials. Those authors suggested that this approach should be complemented with FEM simulations. Din et al. [28] conducted a FEM simulation study in which they applied the 3D Hashin’s theory and Puck’s theory to predict the failure mechanisms caused by adhesive wear. Those authors affirmed that the capability of Puck’s theory to capture the improvement in shear strength, when higher transverse compressive stress is applied, resulted in excellent agreement between the experimental results and the FEM results in a qualitative manner.

## 3. Results

### 3.1. Study Selection and Flow Diagram 

**Figure 1:**
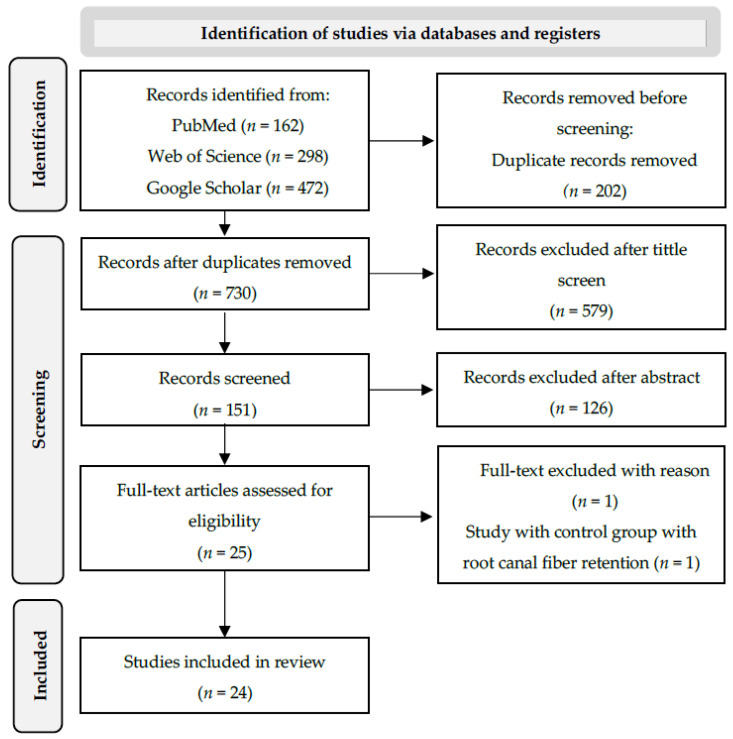
PRISMA flow diagram for systematic reviews [24,25].

A total of 932 preliminary references were identified by searching the electronic databases (Figure 1). After the exclusion of duplicates, 730 articles were selected for title evaluation and 151 articles were submitted to abstract reading and discussion.

After screening, 25 articles were examined at full-text level. One article was excluded for not meeting the inclusion criteria. Twenty-four in vitro studies met the eligibility criteria and were included in this review for qualitative and quantitative data collection. The selected studies and their main details are summarized in Table 2.

### 3.2. Quality Assessment of the Included In Vitro Trials

The detailed assessment of the methodological quality of the studies is shown in Table 3. Twenty-three studies had medium risk of bias. A single study had a high risk of bias [52].

### 3.3. Study Quantitative Results

The highest median value for fracture resistance was exhibited by the control group, healthy teeth, at 1459.9 (962.6 to 2238.92) N, followed by the experimental group, teeth with fiber-reinforced composite restoration, at 976.0 (832.0 to 1834.4) N, and then by the two other control groups, non-fiber composite restoration, at 771.0 (592.2 to 1209.6) N, and unrestored cavity preparation at 386.6 (297.6 to 682.0) N. The experimental and control groups showed differences (Kruskal–Wallis t., *p* ˂ 0.001) for fracture resistance, as presented in Table 4. 

Pairwise comparison of the fracture resistance between the fiber-reinforced composite restorations group and each of the control groups revealed differences (Kruskal–Wallis t.) between the experimental group and the healthy tooth (*p* = 0.048), the non-fiber composite restoration (*p* = 0.008) and the unrestored cavity preparation (*p* < 0.001) groups (Table 5).

## 4. Discussion

The outcomes of 24 in vitro trials, all published in the last 10 years, that had tested and quantified, in Newtons, the fracture resistance of glass and/or polyethylene fibers in extensive posterior composite direct restorations, were evaluated and compared. Fracture resistance mean values were collected and analyzed as independent variables, and their distributions and dispersion were explored. All the in vitro outcomes, the mean values for fracture resistance for the experimental group and for the control groups, showed non-normal distributions (Kolmogorov–Smirnov t., *p* ˂ 0.05). The median values of fracture resistance were calculated for the experimental group, the fiber-reinforced composite restorations, and for each of the control groups in the 24 studies’ protocols, namely, healthy teeth, non-reinforced composite restorations and/or unrestored cavity preparations.

Pairwise comparisons were performed within and between the experimental group and the control groups to detect whether the fracture resistance values for fiber-reinforced composite restorations were similar to those calculated for the healthy teeth, for the non-fiber reinforced composite restorations, and for the unrestored cavity preparations, in posterior extracted human teeth specimens. Based on the results of this systematic quantitative analysis, the null hypothesis was rejected, that is, the median value of fracture resistance for posterior teeth with fiber-reinforced composite restorations differs (*p* ˂ 0.001) from those for healthy teeth, non-fiber-reinforced composite restorations and unrestored cavity preparations. 

The highest median value of fracture resistance, 1459.9 N, was exhibited by the control group, healthy teeth, followed by 976.0 N for the experimental group, the fiber-reinforced composite restorations. Both other control groups, non-fiber composite restoration and unrestored cavity preparation, revealed lower values, at 771.0 N and of 386.6 N, respectively, than either the experimental group or the control group of extracted healthy teeth. 

Posterior teeth restored with fiber-reinforced composites were found to have improved, higher fracture resistance when compared to equivalent teeth restored with non-fiber-reinforced resin composites. This may be attributable to a stress transfer from the polymer matrix of the resin composite within the fiber devices, which have high tensile strength, thus causing lower stress to be transmitted to the remaining tooth structure. Fiber devices may thereby be able to better promote a load spread and distribution within the composite resin restoration [29,30,33,40].

In our analysis, the control group of healthy teeth reported the highest values for fracture resistance when compared to those of fiber-reinforced composite restorations and the other control approaches used in the 24 in vitro research protocols. Those findings emphasize the importance of oral health preventive measures, for preserving the strength of healthy teeth, but also the minimally invasive approach of conservative dentistry, in order to preserve dental hard tissues and tooth surfaces as much as possible during clinical procedures, as structural integrity plays a major role in the tooth’s natural resistance [53]. The loss of tooth structure, whether by extensive cavity preparation, by restorative procedures or by root canal treatments, affects the biomechanical behavior and load–stress distribution within the tooth; this is why restorative procedures and the selection of appropriate materials, in addition to replacing anatomical function and aesthetics, should also aim to reinforce the remaining tooth structure [54].

Compressive and frictional loads can cause fiber bending which takes place in the depth direction (out-of-plane bending) and in the sliding direction (in-plane bending). In-plane and out-of-plane bending contribute to fiber fracture [28]. Friedrich et al. [55] stated that the wear process takes place in a succession of damage mechanisms (or wear cycles). Correspondingly, wear cycles are initiated by matrix wear and fiber sliding wear (also called fiber thinning in the literature). These are followed by fiber fracture and fiber/matrix debonding at the interface.

All 24 of the in vitro studies analyzed with the QUIN tool [26] presented clearly stated aims and detailed the comparison group (positive control, negative control or standard). Most of those studies were adequately specific about the predefined population/problem from which the sample had been selected, and they had detailed explanations of their methodology and process for measurement of the outcomes, as well as details of their statistical analyses and the outcomes based on the predefined objectives. However, a majority of those trials did not present a detailed explanation of sample size calculation, nor did they have information about the number of operators or the training and calibration of operator/s. None of them explained or presented their method for allocation or blinding.

Twenty-two of those studies tested molars and/or premolars with mesial–occlusal–distal cavities (MOD), while one study used mesial–occlusal cavities (MO) [33] and another involved occlusal, MOD and MO cavities [35]. Twenty studies used teeth with root canal treatment and four studies did not perform any root canal treatment on the extracted teeth [29,30,32,40]. Two studies evaluated the effects of using fiberglass [41,44], two studies used fiberglass posts [39,50], two studies used fiberglass and post devices for fiber reinforcement of restorations [33,36], three studies compared glass and polyethylene fibers [40,43,51] and the other fifteen studies tested only polyethylene fibers.

The outcomes of twelve in vitro studies [29,30,31,33,34,40,42,43,48,50,51,52] reported increased fracture resistance mean values for fiber-reinforced composite restorations when compared to groups of non-fiber-reinforced composite restorations. In three of the in vitro trials, the results indicated that fiber-reinforced composite restorations had lower fracture resistance mean values than those of non-fiber-reinforced composite restorations [44,45], and in nine studies the authors reported no significant difference in the fracture resistance mean values of the several groups tested. 

When comparing the results of healthy teeth and those of specimens with fiber-reinforced composite restorations, twelve studies registered higher values of fracture resistance for the control (healthy teeth), but the results of another six studies [36,38,39,42,47,50] did not, significantly, support those findings. Two studies [40,45] reported fracture resistance mean values significantly higher for the group with fiber-reinforced composite restorations than those obtained for healthy teeth. 

In all of the in vitro protocols that defined unrestored cavity preparations as a control group [31,33,34,35,36,41,43,44,45,46,47,50,51,52], the mean values for fracture resistance obtained were always lower than those of the interventional fiber-reinforced composite restorations and those of another control group, the healthy teeth.

In the present study, the diversity of materials used in the 24 reviewed in vitro studies was also taken into account, such as, the fiber type and position procedure, the composite resin filler composition and the clinical consistency (bulk fill, flow or regular), and other types of composite resin tested.

Agrawal et al. [29] and also Balkaya et al. [31] found better results for composite restorations reinforced with polyethylene fibers (Ribbond^®^, bondable reinforcement ribbon, Ribbond Inc., USA) when compared to restorations with EverX Posterior^TM^ (GC, Europe, Street Alsip, IL, USA), a fiber-reinforced composite designed by the manufacturer to be used as dentin replacement in combination with a conventional composite. Hshad et al. [42] and Albar and Khayat [30] also reported increased fracture resistance for teeth restored with polyethylene fibers compared to teeth restored with composite without fibers. Shafiei et al. [34] found the same results when they combined Ribbond^®^, positioned at the background surface, both pulp and axial walls of the cavity preparation, with a nanohybrid composite. Increased values for teeth fracture resistance were also achieved by Rahman et al. [48] using dual polyethylene strips placed at the background wall and the occlusal surfaces of cavity preparations. Similar outcomes were achieved by Singh et al. [52] in their in vitro study, in which the best results corresponded to the tested group that applied both polyethylene fiber and composite resin on the occlusal surface of the posterior restoration. All those results corroborated the findings of previous research that showed using Ribbond^®^ associated with composite resin achieved improvements in the mechanical behavior of the combined tooth/restoration [56,57].

The Ribbond^®^ device is described as a material composed of pre-impregnated, silanized, ultra-high-molecular-weight, plasma-treated polyethylene fibers, with a leno-woven design and a lock-stitch feature allowing, according to some authors, forces to spread throughout the weave without transferring back the stress loads within the composite [58]. According to some authors [29,30,31,34,42,48,52,56,57,58], incorporating polyethylene fibers into composite restorations may provide better results when the fibers are adapted to the inner contours of the remaining tooth substrate, yielding an enhancement of the fracture protection mechanism. The leno weave structure helps to spread the stress over a wider region by providing multiple loading paths so that polymerization shrinkage and occlusal loading stresses are distributed over an extensive surface. Incorporating those devices into a composite restoration is also suggested as an approach to reinforce the tooth by increasing the modulus of elasticity and preventing fractures [16].

In the study of Karzoun et al. [50], teeth restored with composite and fiberglass post devices, horizontally positioned, provided higher fracture resistance than those restored only with composite resin. These findings corroborated those achieved by Tentardini Bainy et al. [33] in their study. The use of fiberglass or fiberglass post positioned in the cavity from the buccal to lingual surfaces, or covering all internal surfaces of the tooth cavity preparations, promoted reinforcement of the cusps, minimizing their deflection effects, as had been previously reported in some studies [59,60].

Sáry et al. [40] evaluated the fracture strength of restorations reinforced with Ribbond^®^, EverX Posterior^TM^ and EverStick^®^NET (GC, Europe) using several restorative approaches. When Ribbond^®^ was transcoronally applied, higher values of fracture resistance were achieved, even slightly higher than those found for the healthy tooth. The fibers’ occlusal positioning in a composite resin restoration leaves them closer to the point of load application, keeps the buccal and lingual cusps together, and promotes higher fracture resistance. Khan et al. [43] also tested the fracture resistance of four different types of fibers. All groups restored with fibers showed higher fracture resistance than those with no fibers within the composite. Also, the groups with EverStick^®^NET and Bioctris^®^ (Bio Composants Médicaux, Saint-Blaise-du-Buis, France) showed higher fracture resistance values compared to Ribbond^®^ and Dentapreg^®^ fiberfill (Dentapreg America Incorporated, Sarasota, FL, USA). Those authors suggested that the fact that both EverStick^®^NET and Bioctris^®^ have unidirectional fibers and a semi-interpenetrating polymer network (semi-IPN) in their composition may improve their chemical bond with the conventional composite. When manual impregnation of fibers is less properly done, that can lead to decreased adhesion between the polyethylene fibers and the composite resin matrix [18]. 

Ozsevik et al. [47] and Garlapati et al. [45], in their studies, compared teeth restored with composite and polyethylene fibers, in this case EverX Posterior^TM^, with non-fiber filler composite restorations. The results revealed fracture resistance values statistically higher in the restorations with EverX Posterior^TM^ than those obtained for the healthy teeth control group. Similar results were found in the study of Eapen et al. [44]. 

EverX Posterior^TM^ is a composite resin with short E-glass fibers and barium glass particles (0.6 to 1.5 mm), randomly oriented within a cross-linked polymer matrix (semi-interpenetrating polymer network, semi IPN), with a total inorganic content of 76% by weight and of 57% by volume. Due to the fibers’ size content, the material is covered by a conventional resin layer, as it does not provide a desirable level for wear resistance or polishing [61]. The better results of EverX Posterior^TM^ can be attributed to the incorporation of those short multidirectional and discontinuous fibers within the resin matrix; they may play a significant role by ameliorating the effects of polymerization shrinkage and marginal microleakage, and supporting the surface composite layer, preventing crack propagation in addition to the load spread [6,20,45,47,61].

However, the studies of Kemaloglu et al. [49], Tekçe et al. [46] and Shah et al. [35] did not find statistical differences between teeth restored with polyethylene fibers and teeth restored with short-fiber-reinforced composites, though fracture resistance values were higher in these two groups than those of teeth restored with composite with no reinforcing fibers. One explanation for those results may be related to the fact that placing the fiber reinforcement within the cavity preparations can be a technically sensitive procedure [35]. Resin composite that is manufactured impregnated with short glass fibers may be clinically easier to apply because it eliminates the need to adapt the fibers separately within the cavities [62]. On the other hand, polyethylene fibers must be impregnated with wetting or bonding adhesive procedures, before their application, creating a critical clinical step. Voids created within the matrix or an excess of residual monomer can affect the interface of the fibers and the composite resin, changing one or both materials’ properties and leading to the restoration’s failure [63]. 

In the research conducted by Jalan et al. [38] and Özüdoğru and Tosun [32], the results showed high fracture resistance for the polyethylene-fiber-reinforced groups, but it was not statistically different from that for healthy teeth or for those restored with conventional composite. These results may differ from other research due to diverse study settings, such as root canal treatment, type of cavity, remaining wall thickness and orientation of the fiber within the cavity [32,38]. The study of Eliguzeloglu Dalkiliç et al. [37] also noted no significant differences for the fracture resistance of composite restorations with or without polyethylene fibers. An explanation for these results may be related to the structural properties of the tested composite material (Estelite Bulk-Fill flow^®^, Tokuyama, Tokyo, Japan), which contained a radical amplified photopolymerization initiator, coupled with camphoroquinone, promoting its polymeric polymerization rates and mechanical properties.

In the studies of Mergulhão et al. [39] and Bahari et al. [36], the fracture resistance of the fiber-reinforced groups did not differ from that of non-fiber-reinforced groups. According to those authors, discrepancies between studies’ outcomes can be attributed to the lack of standardized preparation techniques and testing designs in the several laboratory trials. 

Although the restoration techniques tested in the study of Göktürk et al. [41] increased the fracture resistance of the tested teeth, no significant differences were found between the fracture resistance for teeth restored with composite and that for those with fiber-reinforced composite or hybrid ceramic inlays. In that research, the pre-impregnated E-glass fibers were placed from buccal to lingual position within the cavity and coated with composite. This procedure probably changed the modulus of elasticity of the resin composite, modifying the distribution and transmission of stresses to the residual cavity walls, as also reported by other authors [58].

Glass fibers can be used in different applications, such as engineering, plastic industries, electronic boards and radar housings, as well as in dentistry. Several advantages are offered by fiber-reinforced composites, as they provide suitable aesthetics, non-corrosiveness (metal free), toughness, non-allergenic properties, chairside handling, biocompatibility and the possibility of being custom-made for the specific requirements of many dental applications. As such, its applications include the areas of expertise of prosthodontics (fixed partial dentures), orthodontics (aesthetic retainers), periodontics (splints), endodontics (prefabricated posts) and, as highlighted in this study, direct restorative dentistry [64,65].

A systematic review of in vitro studies was performed in the present work, even though it was initially designed to analyze clinical trial outcomes [24]. However, no clinical data were found for our research question. So, a consensus between the several contradictory laboratory outcomes was achieved. The absence of clear reporting and guidelines data for the in vitro trials was evidenced in the studies reviewed, although testing for the risk of bias was performed. Research protocols such as sample size, specimen allocation, blinding and operator details were not considered or were not described by the authors in the majority of the studies, which may have compromised the quality of some of the outcomes [66,67]. 

Further studies are needed to clarify the influence of some variables, such as the load values and load application angles for mechanical testing, the adhesive strategy, the fiber dimensions and the amount of remaining tooth structure, on the mechanical behavior of restorations with fiber-reinforced composite in extensive posterior teeth cavities. As a future direction, randomized trials should be designed in order to evaluate the clinical, namely, the functional, performance of fiber-reinforced composite restorations in posterior teeth. 

## 5. Conclusions

The outcomes of the 24 in vitro trials published in the last 10 years that tested and measured the fracture resistance of glass and/or polyethylene fibers in extensive posterior composite direct restorations were assessed and compared in this systematic analysis. The values for the fracture resistance of fiber-reinforced composite restorations were higher than those for the controls, namely, the non-fiber-reinforced composite restorations and the unrestored cavity preparations. Fiber-reinforced composites improved the resistance behavior of dental restorations when compared to equivalent teeth restored with composites or unrestored. However, the values for the fracture resistance of the composite and the fiber-reinforced composite restoration combinations were lower than those achieved for healthy teeth.

Resin-based composite with fiber reinforcement can be an appropriate direct restorative approach for coronal restorations of posterior teeth, especially those with extensive cavity preparations in high-stress intra-oral locations. Clinical performance outcomes are needed to support, or invalidate, those in vitro findings.

## Figures and Tables

**Table 1:** Search strategy used in each electronic database.

PubMed		
(Molar [Title/Abstract]) OR (Premolar [Title/Abstract]) OR (Posterior teeth [Title/Abstract])	(Fiber reinforcement [Title/Abstract]) OR (Fiber-reinforced composite dentistry [Title/Abstract]) OR (Fiber-reinforced restoration [Title/Abstract]) OR (Fiber- reinforced composite resin [Title/ Abstract]) OR (Fiber cavity reinforcement [Title/Abstract]) OR (Polyethylene fiber [Title/Abstract]) OR (Glass fiber [Title/Abstract])	(Fracture resistance [Title/ Abstract]) OR (Fracture strength [Title/Abstract]) OR (Fracture resistance fiber-reinforcement composite [Title/Abstract]) OR (in vitro [Title/Abstract])
**Web of Science**		
topic: Molar* OR Bicuspid* OR Premolar* OR Posterior teeth*	topic: Fiber reinforcement* OR Fiber-reinforced composite* OR Fiber- reinforced restoration* OR Fiber-reinforced composite resin* OR Fiber cavity reinforcement* OR Polyethylene fiber* OR Fiberglass*	topic: Fracture resistance* OR Fracture strength* OR fracture resistance fiber-reinforced composite* OR in vitro*
**Google Scholar**		
In title: (Molar OR Premolar OR Posterior teeth)	In title: (Fiber reinforcement OR Fiber-reinforced composite OR Fiber-reinforced restoration OR Fiber cavity reinforcement OR Polyethylene fiber OR Fiberglass)	In title: (Fracture resistance OR Fracture strength OR in vitro)

**Table 2:** Summary of data from the included (*n*= 24) in vitro studies.

First Author, Year	Experimental Group		Control Group	Fracture Resistance Evaluation	Main Conclusion
	Type of Fibers	Application Technique		Mean (SD) ^(1)^ (Newtons)	
Agrawal et al., 2022 [29]	Polyethylene fiber Ribbond^®^ (Ribbond Inc., Seattle, WA, USA)	Gingival and pulpal floor; pulpal floor; vertical on gingival and pulpal floor; fiber chips	Non-fiber- reinforcement composite restorations	Experimental Groups*—G_2_: 1288.8 (186.9); **G_3_**: 976.0 (142.3); **G_4_**: 942.3 (151.5); **G_5_**: 876.3 (165.8)—Control Groups*—G_1_: 588.4 (69.6); **G_6_**: 833.0 (201.1)	Horizontal orientation of fiber on both pulpal and gingival floor of MOD cavities gives the highest fracture resistance
Albar et al., 2022 [30]	Polyethylene fiber Ribbond^®^ (Ribbond Inc.)	Axial wall of the proximal cavity; gingival floor of the proximal cavity; axial wall and pulpal/gingival floor of the proximal cavity	Non-fiber- reinforcement composite restorations	Experimental Groups*—G_2_: 422.1 (14.9); **G_3_**: 409.0 (15.9); **G_4_**: 446.2 (12.9)—Control Group*—G_1_: 390.2 (10.4)	The reinforcement of direct composite resin restorations with polyethylene fibers increased the fracture resistance of the restorations in comparison with non-reinforced restorations
Balkaya et al., 2022 [31]	Polyethylene fiber Ribbond^®^ (Ribbond Inc.)	Buccal, lingual and pulpal walls	Positive control (PC) unrestored cavity; negative control (NC) healthy teeth	Experimental Groups*—G_7_: 601.0 (133.0); **G_8_**: 658.0 (116.0) Control Groups*—NC: 952.0 (111.0); **PC**: 219.0 (48.0); **non-fiber-reinforcement composite**: **G_3_**: 440.0 (102.0); **G_4_**: 447.0 (101.0); **G_5_**: 459.0 (126.0); **G_6_**: 464.0 (115.0)	Ribbond in combination with composite resin enhanced the fracture resistance of teeth
Özüdoğru et al., 2022 [32]	Polyethylene fiber Ribbond^®^ (Ribbond Inc.)	Buccal and lingual walls and fiber placed in circumferential way	Healthy teeth (HT)	Experimental Groups*—G_3_: 2602.1 (126.2); **G_4_**: 2805.7 (125.9) Control Groups*—HT: 2710.4 (171.2); **non-fiber**- **reinforcement composite: G_2_**: 2312.5 (112.0)	Polyethylene fiber reinforcement did not affect the fracture resistance of composite resin restorations
Tentardini Bainy et al., 2021 [33]	Fiberglass post Reforpost^®^ (Angelus, Los Angeles, CA, USA) Fiberglass Interlig^®^ (Angelus)	Horizontal transfixation on buccal and palatal walls; Placed in circumferential way	Positive control (PC) healthy teeth; negative control (NC) unrestored cavity	Experimental Groups*—G_4_: 2256.0 (289.2); **G_5_**: 2493.0 (364.0) Control Groups*—PC: 3563.0 (780.7); **NC**: 1001.0 (237.6); **non-fiber-reinforcement composite: G_3_**: 1689.0 (280.7)	The fiberglass, regardless of composition, increases the fracture resistance of endodontically treated teeth
Shafiei et al., 2021 [34]	Polyethylene fiber Ribbond^®^ (Ribbond Inc.)	Buccal, lingual and pulpal walls	Positive control (PC) unrestored cavity; negative control (NC) healthy teeth	Experimental Groups*—G_6_: 858.0 (215.0); **G_7_**: 529.0 (124.0); **G_8_**: 802.0 (201.0)—Control Groups*—NC: 1204.0 (252.0); **PC**: 352.0 (143.0); **non-fiber-reinforcement composite**: **G_3_**: 579.0 (114.0); **G_4_**: 596.0 (138.0); **G_5_**: 624.0 (182.0)	The effect of fiber on fracture resistance depended on the type of composite resin; the highest reinforcing effect was obtained in the conventional composite resin and fiber
Shah et al., 2020 [35]	Polyethylene fiber Ribbond^®^ (Ribbond Inc.)	Buccal, lingual and pulpal walls	Positive control (PC) healthy teeth; negative control (NC) unrestored cavity	Experimental Groups*—G_4_: 797.9 (17.7); **G_5_**: 834.7 (26.3); **G_6_**: 843.9 (39.8)—Control Groups*—PC: 1207.4 (90.6); **NC**: 669.6 (15.0); **non-fiber-reinforcement composite**: **G_1_**: 879.9 (36.3); **G_2_**_:_ 873.6 (38.3); **G_3_**: 922.6 (23.3); **G_7_**_:_ 697.7 (34.9); **G_8_**_:_ 705.4 (18.5); **G_9_**_:_ 713.0 (11.6)	Fiber-reinforced composites when used in different cavity configurations of endodontically treated premolar yielded similar results
Bahari et al., 2019 [36]	Fiberglass Interlig^®^ (Angelus) Fiberglass post Reforpost^®^ (Angelus) Fiberglass post and fiberglass	Buccal and lingual walls; Horizontal transfixation on buccal and palatal walls; Horizontal on buccal and palatal walls and occlusal position	Positive control (PC) healthy teeth; negative control (NC) unrestored cavity	Experimental Groups*—G_4_: 1122.1 (231.6); **G_5_**: 1023.3 (295.5); **G_6_**: 1097.5 (256.0)—Control Groups*—PC: 1073.6 (245.1); **NC**: 461.8 (136.2); **non-fiber-reinforcement** **composite**: **G_3_**: 1103.5 (378.4)	Fiber reinforcement has no additional reinforcing effect on fracture strength of composite-resin-restored endodontically treated maxillary premolars
Eliguzeloglu Dalkiliç et al., 2019 [37]	Polyethylene fiber Ribbond^®^ (Ribbond Inc.)	Buccal, lingual and pulpal walls; fiber in base of cavity and occlusal position	Healthy teeth (HT)	Experimental Groups*—G_4_: 818.9 (166.1); **G_5_**: 821.9 (226.3); **G_7_**: 803.3 (78.1); **G_8_**: 832.0 (209.2)—Control Groups*—HT_1_: 1351.4 (238.8); **HT_2_**: 1210.0 (318.5); **non-fiber-reinforcement composite: G_3_**: 736.8 (116.4); G_6_: 788.7 (210.5)	Fiber insertion with different techniques did not increase the fracture strength of teeth restored with bulk-fill composites
Jalan et al., 2019 [38]	Polyethylene fiber Ribbond^®^ (Ribbond Inc.)	Buccal, lingual and pulpal walls; fiber on the occlusal surface	Healthy teeth (HT)	Experimental Groups*—G_3_: 1114.5 (429.9); **G_4_**: 725.9 (118.7) Control Groups*—HT: 914.3 (695.2); **non-fiber***—* **reinforcement composite: G_2_**: 984.6 (403.4)	Fiber reinforcement in base of cavity might be an alternate technique for a permanent restoration after root canal treatment
Mergulhão et al., 2019 [39]	Fiberglass post White Post DC^™^ (FGM, Santa Catarina, Brazil)	Horizontal on the buccal and palatal walls	Healthy teeth (HT)	Experimental Group*—G_3_: 934.5 (233.6)—Control Groups* HT: 949.6 (331.5); **non-fiber-reinforcement composite**: **G_2_**: 999.6 (352.50); **G_4_**: 771.0 (147.4)	Endodontically treated maxillary premolars restored with conventional composite resin with or without horizontal fiber post, bulk-fill composite or ceramic inlay showed fracture resistance like that of sound teeth
Sáry et al., 2019 [40]	Polyethylene fiber Ribbond^®^ (Ribbond Inc.) Fiberglass EverStick NET^®^ (GC Corporation, Tokyo, Japan)	Buccal/lingual in base of cavity; on the top; as an occlusal splint; circumferentially or transcoronally Buccal/lingual in base of cavity; on the top; as an occlusal splint or circumferentially	Healthy teeth (HT)	Experimental Groups*—G_3_: 1122.2 (440.0); **G_4_**: 1408.6 (314.5); **G_5_**: 1925.6 (792.6); **G_6_**: 2067.3 (535.8); **G_7_**: 1834.4 (578.5); **G_8_**: 2022.0 (771.4); **G_9_**: 2129.2 (629.7); **G_10_**: 1906.9 (538.0); **G_11_**: 2484.8 (682.9)—Control Groups*—HT: 2266.3 (601.1); **non-fiber-reinforcement composite**: **G_1_**: 1629.4 (503.1); **G_2_**: 1746.2 (467.5)	Incorporating polyethylene or a combination of short and bidirectional glass fibers in certain positions in direct restorations seems to be able to restore the fracture resistance of sound molar teeth
Göktürk et al., 2018 [41]	Fiberglass Interlig^®^ (Angelus)	Buccal and lingual walls	Positive control (PC) healthy teeth; negative control (NC) unrestored cavity	Experimental Group*—G_4_: 367.1 (82.9)—Control Groups*—PC: 742.0 (245.4); **NC**: 192.1 (59.3); **non-fiber-** **reinforcement composite: G_3_**: 355.8 (103.9)	All the restoration techniques increased the fracture resistance of teeth; there were no significant differences between the fracture resistance values of the groups that underwent different restorations
Hshad et al., 2018 [42]	Polyethylene fiber Ribbond^®^ (Ribbond Inc.)	Buccal, lingual and pulpal walls	Healthy teeth (HT)	Experimental Group*—G_3_: 1951.6 (330.9) Control Groups*—HT: 2156.7 (628.0); **non-fiber**- **reinforcement composite: G_2_**: 1315.8 (352.3); **G_4_**: 1445.3 (506.1)	Polyethylene fiber considerably increases the fracture resistance of mandibular premolar teeth with MOD cavities restored with composite
Khan et al., 2018 [43]	Polyethylene fiber Ribbond^®^ (Ribbond Inc.); Fiberglass EverStick^®^ (GC Corporation); Dentapreg^®^ (Advanced Dental Materials); Bioctris^®^ (Bio Composants Medicaux, Saint-Blaise-du-Buis, France)	Buccal, lingual and pulpal walls	Positive control (PC) healthy teeth; negative control (NC) unrestored cavity	Experimental Groups*—G_4_: 959.2 (128.6); **G_5_**: 1433.1 (98.5); **G_6_**: 979.1 (124.2); **G_7_**: 1480.20 (102.9)—Control Groups*— **PC**: 1677.0 (155.1); **NC**: 352.5 (32.7); **non-fiber**- **reinforcement composite: G_3_**: 775.1 (101.9)	All the groups restored with fiber displayed higher fracture resistance than the group restored with only composite resin; E glass fibers demonstrated highest fracture resistance and hence can be preferred over other fiber types
Eapen et al., 2017 [44]	Fiberglass Interlig^®^ (Angelus)	Buccal, lingual and pulpal walls	Positive control (PC) unrestored cavity; negative control (NC) healthy teeth	Experimental Group*—G_5_: 404.1 (94.2)—Control Groups*—PC: 233.8 (26.4); **NC**: 842.5 (294.4); **non-fiber**- **reinforcement composite: G_3_**: 434.5 (174.3); G_4_: 465.13 (159.3); G_6_: 712.8 (79.8)	Short-fiber-reinforced composite can be used as a direct core buildup material that can effectively resist heavy occlusal forces against fracture and may reinforce the remaining tooth structure
Garlapati et al., 2017 [45]	Polyethylene fiber Ribbond^®^ (Ribbond Inc.)	Buccal, lingual and pulpal walls	Positive control (PC) healthy teeth; negative control (NC) Unrestored cavity	Experimental Group*—G_4_: 1716.7 (199.5)—Control Groups*—PC: 1568.40 (221.7); **NC**: 891.00 (50.1); **non-fiber**- **reinforcement composite: G_3_**: 1418.3 (168.7); **G_5_**: 1994.80 (254.2)	Endodontically treated teeth restored with EverX Posterior fiber-reinforced composite showed superior fracture resistance
Tekçe et al., 2017 [46]	Polyethylene fiber Ribbond^®^ (Ribbond Inc.)	Buccal, lingual and pulpal walls	Positive control (PC) Healthy teeth Negative control (NC) unrestored cavity	Experimental Groups*—G_1_: 2254.1 (324.8); **G_2_** 2228.6 (409.3); **G_3_**: 2007.40(495.6); **G_4_**: 1938.2 (199.7)—Control Groups*—PC: 2910.3 (361.0); **NC**: 719.30 (108.6); **non-fiber**- **reinforcement composite: G_5_**: 2142.9 (411.5)	Ribbond- or short-fiber-reinforced composites modestly increased the fracture resistance of unfilled teeth; polyethylene-fiber- reinforced composite groups displayed similar fracture resistance results to those of the EverX Posterior group
Ozsevik et al., 2016 [47]	Polyethylene fiber Ribbond^®^ (Ribbond Inc.)	Buccal, lingual and pulpal walls	Positive control (PC) healthy teeth; negative control (NC) unrestored cavity	Experimental Group*—G_4_: 1958.0 (362.9)—Control Groups*—PC: 2859.5 (551.2); **NC**: 318.9 (108.6); **non-fiber**- **reinforcement composite: G_3_**: 1489.5 (505.0); **G_5_**: 2550.7 (586.1)	Fiber-reinforced composite under composite restorations resulted in fracture resistance similar to that of intact teeth; furthermore, it reinforced root-filled teeth more than composite alone and Ribbond and composite restorations
Rahman et al., 2016 [48]	Polyethylene fiber Ribbond^®^ (Ribbond Inc.)	Buccal/lingual on the occlusal surface; buccal/lingual in base of cavity; buccal/lingual in base of cavity and fiber on the occlusal surface	Non-fiber- reinforcement composite restorations	Experimental Groups*—G_2_: 1236.8 (83.4); **G_2_**: 879.3 (98.2); **G_2_**: 1482.0 (74.5)—Control Group*—G_1_: 653.4 (74.0)	Polyethylene fiber inserted over or under the restoration significantly increased the fracture resistance of the root-canal-treated teeth and maximum fracture resistance was observed with dual-fiber technique
Kemaloglu et al., 2015 [49]	Polyethylene fiber Ribbond^®^ (Ribbond Inc.)	Buccal, lingual and pulpal walls	Non-fiber- reinforcement composite restorations	Experimental Group*—G_2_: 919.8 (47.6)—Control Groups*—G_1_: 823.3 (34.0); **G_3_**: 889.4 (72.8); **G_4_**: 817.1 (60.8)	Fiber-reinforcement improved the fracture resistance of teeth with large MOD cavities treated endodontically
Karzoun et al., 2015 [50]	Fiberglass post White Post DC^™^ (FGM)	Fiber post through the buccal and lingual walls	Positive control (PC) healthy teeth; negative control (NC) unrestored cavity	Experimental Groups*—G_4_: 961.3 (245.2); **G_5_**: 656.0 (139.4)—Control Groups*—PC: 994.5 (147.3); **NC**: 411.8 (104.0); **non-fiber-reinforcement composite: G_3_**: 482.1 (72.9)	Using a horizontal fiberglass post to restore endodontically treated MOD cavities increased the fracture resistance of the restoration tooth significantly
Khan et al., 2013 [51]	Polyethylene fiber Ribbond^®^ (Ribbond Inc.); Fiberglass Vectris^®^ (Ivoclar, Tokyo, Japan)	Buccal, lingual and pulpal walls	Positive control (PC) healthy teeth; negative control (NC) unrestored cavity	Experimental Groups*—G_5_: 958.6 (162.7); **G_6_**: 913.2 (151.3) Control Groups*—**PC**: 1598.8 (168.3); **NC**: 393.7 (24.4); **non-fiber-reinforcement composite: G_3_**: 729.3 (168.0); **G_4_**: 699.7 (114.5)	Polyethylene and fiberglass under MOD composite restorations significantly increased fracture strength with no statistical difference between the two groups
Singh et al., 2013 [52]	Polyethylene fiber Ribbond^®^ (Ribbond Inc.)	Fiber strip in bucco-lingually oriented groove on the restoration’s occlusal surface; fiber on the buccal, lingual and pulpal walls	Positive control (PC) healthy teeth; negative control (NC) unrestored cavity	Experimental Groups*—G_3_: 1236.8 (83.4); **G_4_**: 879.3 (98.2)—Control Groups*—PC: 1674.0 (99.7); **NC**: 379.6 (34.9); **non-fiber-reinforcement composite: G_2_**: 653.4 (74.0)	Polyethylene fiber inserted over or under the restoration significantly increased the fracture strength of the root-canal-treated teeth

^(1)^ SD: standard deviation; * groups’ names as represented in the in vitro studies; G: group; MOD: Mesial–Occlusal–Distal.

**Table 3:** Quality assessment of the in vitro studies using the QUIN tool [26].

First Author, Year	1. Clearly Stated Aims/Objectives	2. Sample Size Calculation	3. Explanation of Sampling Technique	4. Comparison Group	5. Methodology	6. Operator Details	7. Randomization	8. Method of Measurement of Outcome	9. Outcome Assessor Details	10. Blinding	11. Statistical Analysis	12. Presentation of Results	Risk of Bias *
Agrawal et al., 2022 [29]	2	0	2	2	2	0	0	2	0	0	2	2	Medium
Albar et al., 2022 [30]	2	2	2	2	2	0	0	2	0	0	2	2	Medium
Balkaya et al., 2022 [31]	2	2	2	2	2	0	0	2	0	0	2	2	Medium
Özüdoğru et al., 2022 [32]	2	0	2	2	2	1	0	2	0	0	2	2	Medium
Tentardini Bainy et al., 2021 [33]	2	2	2	2	2	0	0	2	0	0	2	2	Medium
Shafiei et al., 2021 [34]	2	0	2	2	2	0	0	2	0	0	2	2	Medium
Shah et al., 2020 [35]	2	0	2	2	2	0	0	2	0	0	2	2	Medium
Bahari et al., 2019 [36]	2	2	2	2	2	0	0	2	0	0	2	2	Medium
Eliguzeloglu Dalkiliç et al., 2019 [37]	2	0	2	2	2	0	0	2	0	0	2	2	Medium
Jalan et al., 2019 [38]	2	0	2	2	2	0	0	2	0	0	1	1	Medium
Mergulhão et al., 2019 [39]	2	0	2	2	2	0	0	2	0	0	2	2	Medium
Sáry et al., 2019 [40]	2	0	2	2	2	1	0	2	0	0	2	2	Medium
Göktürk et al., 2018 [41]	2	0	2	2	2	1	0	2	0	0	2	2	Medium
Hshad et al., 2018 [42]	2	0	2	2	2	0	0	2	0	0	2	2	Medium
Khan et al., 2018 [43]	2	0	2	2	2	0	0	2	0	0	2	2	Medium
Eapen et al., 2017 [44]	2	0	2	2	2	0	0	2	0	0	2	2	Medium
Garlapati et al., 2017 [45]	2	0	2	2	2	0	0	2	0	0	1	2	Medium
Tekçe et al., 2017 [46]	2	0	2	2	2	0	0	2	0	0	2	2	Medium
Ozsevik et al., 2016 [47]	2	2	2	2	2	0	0	2	0	0	2	2	Medium
Rahman et al., 2016 [48]	2	0	2	2	2	0	0	2	0	0	1	2	Medium
Kemaloglu et al., 2015 [49]	2	0	2	2	2	0	0	2	0	0	1	2	Medium
Karzoun et al., 2015 [50]	2	0	2	2	2	1	0	2	0	0	2	2	Medium
Khan et al., 2013 [51]	2	0	2	2	2	0	0	2	0	0	1	2	Medium
Singh et al., 2013 [52]	2	0	1	2	1	0	0	1	0	0	1	1	High

* Score: adequately specified = 2; inadequately specified = 1; not specified (NS) = 0; not applicable (NA). Final Score: total score×100/2 × number of criteria applicable. >70% = low risk of bias; 50–70% = medium risk of bias; and <50% = high risk of bias.

**Table 4:** Fracture resistance (N) obtained for the experimental group and for each the control groups.

Experimental (Exp) and Control (C) Groups	Overall Fracture Resistance (N)			*p*-Value ^(3)^
	*n* ^(1)^	Median Value	IQR ^(2)^ Values	
**Exp**: Fiber-reinforced composite restoration	55	976.0	832.0–1834.4	*˂*0.001
**C**: Healthy teeth	20	1459.9	962.6–2238.9	
**C**: Non-fiber-reinforced composite restoration	45	771.0	592.2–1209.6	
**C**: Unrestored cavity preparation	14	386.6	297.6–682.0	

^(1)^ *n*: number of posterior teeth per group. ^(2)^ IQR: interquartile range (Q_1_–Q_3_). ^(3)^ Kruskal–Wallis test (*p* < 0.05).

**Table 5:** Pairwise comparison between the experimental group and each of the control groups.

Pairwise Comparisons of Experimental and Each Control Groups		
Experimental Group Sample 1 ^(1)^	Control Groups Sample 2 ^(1)^	*p*-Value ^(2)^
Fiber-reinforced composite restorations	Healthy teeth	0.048
	Non-fiber-reinforced composite restorations	0.008
	Unrestored cavity preparations	<0.001

^(1)^ Each line tested the null hypothesis that the distributions of Sample 1 and Sample 2 are equal (95%). ^(2)^ Kruskal–Wallis test. Significance values were adjusted by Bonferroni correction.

## Data Availability

Not applicable.

## References

[1] Gaeta C., Marruganti C., Mignosa E., Franciosi G., Ferrari E., Grandini S. (2021). Influence of Methodological Variables on Fracture Strength Tests Results of Premolars with Different Number of Residual Walls. A Systematic Review with Meta-Analysis. Dent. J..

[2] Vetromilla B.M., Opdam N.J., Leida F.L., Sarkis-Onofre R., Demarco F.F., Van der Loo M.P.J., Cenci M.S., Pereira-Cenci T. (2020). Treatment options for large posterior restorations: A systematic review and network meta-analysis. J. Am. Dent. Assoc..

[3] Nam S.H., Chang H.S., Min K.S., Lee Y., Cho H.W., Bae J.M. (2010). Effect of the number of residual walls on fracture resistances, failure patterns, and photoelasticity of simulated premolars restored with or without fiber-reinforced composite posts. J. Endod..

[4] Ferracane J.L., Lawson N.C. (2021). Probing the hierarchy of evidence to identify the best strategy for placing class II dental composite restorations using current materials. J. Esthet. Restor. Dent..

[5] Da Rosa Rodolpho P.A., Rodolfo B., Collares K., Correa M.B., Demarco F.F., Opdam N.J.M., Cenci M.S., Moraes R.R. (2022). Clinical performance of posterior resin composite restorations after up to 33 years. Dent. Mater..

[6] Lassila L., Keulemans F., Säilynoja E., Vallittu P.K., Garoushi S. (2018). Mechanical properties and fracture behavior of flowable fiber reinforced composite restorations. Dent. Mater..

[7] Chai H. (2023). On the fracture behavior of molar teeth with MOD cavity preparation. J. Mech. Behav. Biomed. Mater..

[8] Fráter M., Sáry T., Vincze-Bandi E., Volom A., Braunitzer G., Szabó P.B., Garoushi S., Forster A. (2021). Fracture Behavior of Short Fiber-Reinforced Direct Restorations in Large MOD Cavities. Polymers.

[9] Scribante A., Vallittu P.K., Özcan M. (2018). Fiber-Reinforced Composites for Dental Applications. BioMed Res. Int..

[10] Vallittu P.K. (2015). High-aspect ratio fillers: Fiber-reinforced composites and their anisotropic properties. Dent. Mater..

[11] Vallittu P.K. (2018). An overview of development and status of fiber-reinforced composites as dental and medical biomaterials. Acta Biomater. Odontol. Scand..

[12] Scribante A., Vallittu P.K., Özcan M., Lassila L.V.J., Gandini P., Sfondrini M.F. (2018). Travel beyond Clinical Uses of Fiber Reinforced Composites (FRCs) in Dentistry: A Review of Past Employments, Present Applications, and Future Perspectives. BioMed Res. Int..

[13] Rana M.H., Shaik S., Hameed M.S., Al-Saleh S., AlHamdan E.M., Alshahrani A., Alqahtani A., Albaqawi A.H., Vohra F., Abduljabbar T. (2021). Influence of Dental Glass Fibers and Orthopedic Mesh on the Failure Loads of Polymethyl Methacrylate Denture Base Resin. Polymers.

[14] Miao Y., Liu T., Lee W., Fei X., Jiang G., Jiang Y. (2016). Fracture resistance of palatal cusps defective premolars restored with polyethylene fiber and composite resin. Dent. Mater. J.

[15] Aggarwal V., Singla M., Miglani S., Sharma V., Kohli S. (2018). Effect of polyethylene fiber reinforcement on marginal adaptation of composite resin in Class II preparations. Gen. Dent..

[16] Deliperi S., Alleman D., Rudo D. (2017). Stress-reduced Direct Composites for the Restoration of Structurally Compromised Teeth: Fiber Design According to the ‘Wallpapering’ Technique. Oper. Dent..

[17] Mangoush E., Säilynoja E., Prinssi R., Lassila L., Vallittu P., Garoushi S. (2017). Comparative evaluation between glass and polyethylene fiber reinforced composites: A review of the current literature. J. Clin. Exp. Dent..

[18] Mangoush E., Garoushi S., Lassila L., Vallittu P.K., Säilynoja E. (2021). Effect of Fiber Reinforcement Type on the Performance of Large Posterior Restorations: A Review of In Vitro Studies. Polymers.

[19] Jakab A., Volom A., Sáry T., Vincze-Bandi E., Braunitzer G., Alleman D., Garoushi S., Fráter M. (2022). Mechanical Performance of Direct Restorative Techniques Utilizing Long Fibers for “Horizontal Splinting” to Reinforce Deep MOD Cavities—An Updated Literature Review. Polymers.

[20] Albar N., Khayat W. (2023). Fracture Load of Mesio–Occluso–Distal Composite Restorations Performed with Different Reinforcement Techniques: An In Vitro Study. Polymers.

[21] Preethikaharshini J., Naresh K., Rajeshkumar G., Arumugaprabu V., Khan M.A., Khan K.A. (2022). Review of advanced techniques for manufacturing biocomposites: Non-destructive evaluation and artificial intelligence-assisted modeling. J. Mater. Sci..

[22] Joffre T., Isaksson P., Dumont P.J.J., Du Roscoat S.R., Sticko S., Orgéas L., Gamstedt E.K. (2016). A Method to Measure Moisture Induced Swelling Properties of a Single Wood Cell. Exp. Mech..

[23] Strohrmann K., Hajek M. (2019). Bilinear approach to tensile properties of flax composites in finite element analyses. J. Mater. Sci..

[24] Page M.J., McKenzie J.E., Bossuyt P.M., Boutron I., Hoffmann T.C., Mulrow C.D., Shamseer L., Tetzlaff J.M., Akl E.A., Brennan S.E. (2021). The PRISMA 2020 statement: An updated guideline for reporting systematic reviews. BMJ.

[25] Equator Network. https://www.equator-network.org/reporting-guidelines/prisma/.

[26] Sheth V.H., Shah N.P., Jain R., Bhanushali N., Bhatnagar V. (2022). Development and validation of a risk-of-bias tool for assessing in vitro studies conducted in dentistry: The QUIN. J. Prosthet. Dent..

[27] Mzali S., Elwasli F., Mkaddem A., Mezlini S. (2018). A micromechanical scratch model to investigate wear mechanisms in UD-GFRP composites. Mech. Ind..

[28] Din I.U., Panier S., Hao P., Franz G., Bijwe J., Hui L. (2019). Finite element modeling of indentation and adhesive wear in sliding of carbon fiber reinforced thermoplastic polymer against metallic counterpart. Tribol. Int..

[29] Agrawal V.S., Shah A., Kapoor S. (2022). Effect of fiber orientation and placement on fracture resistance of large class II mesio-occluso-distal cavities in maxillary premolars: An in vitro study. J. Conserv. Dent..

[30] Albar N.H.M., Khayat W.F. (2022). Evaluation of Fracture Strength of Fiber-Reinforced Direct Composite Resin Restorations: An In Vitro Study. Polymers.

[31] Balkaya H., Topçuoğlu H.S., Demirbuga S., Kafdağ Ö., Topçuoğlu G. (2022). Effect of different coronal restorations on the fracture resistance of teeth with simulated regenerative endodontic treatment: An in vitro study. Aust. Endod. J..

[32] Özüdoğru S., Tosun G. (2022). Evaluation of Microleakage and Fatigue Behaviour of Several Fiber Application Techniques in Composite Restorations. Ann. Dent. Spec..

[33] Tentardini Bainy P., Melara R., Burnett Junior L.H., Fontoura de Melo T.A. (2021). Effect of glass fiber on the restorative procedure in relation to fracture strength of endodontically treated molars. G. Ital. Endodon..

[34] Shafiei F., Dehghanian P., Ghaderi N., Doozandeh M. (2021). Fracture resistance of endodontically treated premolars restored with bulk-fill composite resins: The effect of fiber reinforcement. Dent. Res. J..

[35] Shah S., Shilpa-Jain D.P., Velmurugan N., Sooriaprakas C., Krithikadatta J. (2020). Performance of fibre reinforced composite as a post-endodontic restoration on different endodontic cavity designs- an in-vitro study. J. Mech. Behav. Biomed. Mater..

[36] Bahari M., Mohammadi N., Kimyai S., Kahnamoui M.A., Vahedpour H., Torkani MA M., Oskoee A.S. (2019). Effect of Different Fiber Reinforcement Strategies on the Fracture Strength of Composite Resin Restored Endodontically Treated Premolars. Pesqui. Bras. Odontopediatr. Clín. Integr..

[37] Eliguzeloglu Dalkiliç E., Kazak M., Hisarbeyli D., Fildisi M.A., Donmez N., Deniz Arısu H. (2019). Can Fiber Application Affect the Fracture Strength of Endodontically Treated Teeth Restored with a Low Viscosity Bulk-Fill Composite?. BioMed Res. Int..

[38] Jalan R., Merwade S., Kumar K.N., Naik S., Brigit B., Jain N. (2019). A Comparative Study to Evaluate the Fracture Resistance of Endodontically Treated Maxillary Premolar Teeth with MOD Cavity Preparation, Restored with Composite Resin and Different Positions of Polyethylene Fibre Insertion-An In Vitro Study. J. Adv. Med. Dent. Scie. Res..

[39] Mergulhão V., de Mendonça L., de Albuquerque M., Braz R. (2019). Fracture Resistance of Endodontically Treated Maxillary Premolars Restored with Different Methods. Oper. Dent..

[40] Sáry T., Garoushi S., Braunitzer G., Alleman D., Volom A., Fráter M. (2019). Fracture behaviour of MOD restorations reinforced by various fibre-reinforced techniques—An in vitro study. J. Mech. Behav. Biomed. Mater..

[41] Göktürk H., Karaarslan E.Ş., Tekin E., Hologlu B., Sarıkaya I. (2018). The effect of the different restorations on fracture resistance of root-filled premolars. BMC Oral Health.

[42] Hshad M.E., Dalkılıç E.E., Ozturk G.C., Dogruer I., Koray F. (2018). Influence of Different Restoration Techniques on Fracture Resistance of Root-filled Teeth: In Vitro Investigation. Oper. Dent..

[43] Khan S.I., Ramachandran A., Alfadley A., Baskaradoss J.K. (2018). Ex vivo fracture resistance of teeth restored with glass and fiberreinforced composite resin. J. Mech. Behav. Biomed. Mater..

[44] Eapen A.M., Amirtharaj L.V., Sanjeev K., Mahalaxmi S. (2017). Fracture Resistance of Endodontically Treated Teeth Restored with 2 Different Fiber-reinforced Composite and 2 Conventional Composite Resin Core Buildup Materials: An In Vitro Study. J. Endod..

[45] Garlapati T.G., Krithikadatta J., Natanasabapathy V. (2017). Fracture resistance of endodontically treated teeth restored with short fiber composite used as a core material-An in vitro study. J. Prosthodont. Res..

[46] Tekce N., Pala K., Tuncer S., Demirci M., Serim M.E. (2017). Influence of polymerisation method and type of fibre on fracture strength of endodontically treated teeth. Aust. Endod. J..

[47] Ozsevik A.S., Yildirim C., Aydin U., Culha E., Surmelioglu D. (2015). Effect of fibre-reinforced composite on the fracture resistance of endodontically treated teeth. Aust. Endod. J..

[48] Rahman H., Singh S., Chandra A., Chandra R., Tripathi S. (2016). Evaluation of fracture resistance of endodontically treated teeth restored with composite resin along with fibre insertion in different positions in vitro. Aust. Endod. J..

[49] Kemaloglu H., Kaval M.E., Turkun M., Kurt S.M. (2015). Effect of novel restoration techniques on the fracture resistance of teeth treated endodontically: An in vitro study. Dent. Mater. J..

[50] Karzoun W., Abdulkarim A., Samran A., Kern M. (2015). Fracture Strength of Endodontically Treated Maxillary Premolars Supported by a Horizontal Glass Fiber Post: An In Vitro Study. J. Endod..

[51] Khan S.I., Anupama R., Deepalakshmi M., Kumar K.S. (2013). Effect of two different types of fibers on the fracture resistance of endodontically treated molars restored with composite resin. J. Adhes. Dent..

[52] Singh S., Chandra A., Tikku A.P., Verma P. (2013). A comparative evaluation of different restorative technique using polyethylene fibre in reinforcing the root-filled teeth: An in vitro study. J. Res. Dent..

[53] Zhang Y.R., Du W., Zhou X.D., Yu H.Y. (2014). Review of research on the mechanical properties of the human tooth. Int. J. Oral Sci..

[54] Neto M.A., Roseiro L., Messias A., Falacho R.I., Palma P.J., Amaro A.M. (2021). Influence of Cavity Geometry on the Fracture Strength of Dental Restorations: Finite Element Study. Appl. Sci..

[55] Friedrich K., Fakirov S., Zhang Z. (2005). Polymer Composites: From Nano-to-Macro-Scale.

[56] Belli S., Erdemir A., Ozcopur M., Eskitascioglu G. (2005). The effect of fibre insertion on fracture resistance of root filled molar teeth with MOD preparations restored with composite. Int. Endod. J..

[57] Ayad M.F., Maghrabi A.A., García-Godoy F. (2010). Resin composite polyethylene fiber reinforcement: Effect on fracture resistance of weakened marginal ridges. Am. J. Dent..

[58] Belli S., Erdemir A., Yildirim C. (2006). Reinforcement effect of polyethylene fibre in root-filled teeth: Comparison of two restorationtechniques. Int. Endod. J..

[59] Beltrão M.C., Spohr A.M., Oshima H.M., Mota E.G., Burnett L.H. (2009). Fracture strength of endodontically treated molars transfixed horizontally by a fiber glass post. Am. J. Dent..

[60] Oskoee P.A., Ajami A.A., Navimipour E.J., Oskoee S.S., Sadjadi J. (2009). The effect of three composite fiber insertion techniques on fracture resistance of root-filled teeth. J. Endod..

[61] Garoushi S., Säilynoja E., Vallittu P.K., Lassila L. (2013). Physical properties and depth of cure of a new short fiber reinforced composite. Dent. Mater..

[62] Forster A., Braunitzer G., Tóth M., Szabó B.P., Fráter M. (2019). In Vitro Fracture Resistance of Adhesively Restored Molar Teeth with Different MOD Cavity Dimensions: Fracture Resistance of Adhesively Restored MOD Cavities. J. Prosthodont..

[63] Sadr A., Bakhtiari B., Hayashi J., Luong M.N., Chen Y.W., Chyz G., Chan D., Tagami J. (2020). Effects of fiber reinforcement on adaptation and bond strength of a bulk-fill composite in deep preparations. Dent. Mater..

[64] Scotti N., Forniglia A., Tempesta R.M., Comba A., Saratti C.M., Pasqualini D., Alovisi M., Berutti E. (2016). Effects of fiber-glassreinforced composite restorations on fracture resistance and failure mode of endodontically treated molars. J. Dent..

[65] Safwat E.M., Khater A.G.A., Abd-Elsatar A.G., Khater G.A. (2021). Glass fiber-reinforced composites in dentistry. Bull. Natl. Res. Cent..

[66] Krithikadatta J., Gopikrishna V., Datta M. (2014). CRIS Guidelines (Checklist for Reporting In-vitro Studies): A concept note on the need for standardized guidelines for improving quality and transparency in reporting in-vitro studies in experimental dental research. J. Conserv. Dent..

[67] Hammel C., Pandis N., Pieper D., Faggion C.M. (2022). Methodological assessment of systematic reviews of in-vitro dental studies. BMC Med. Res. Methodol..

